# Mesenchymal Stem Cells Combined with Tissue Fusion Technology Promoted Wound Healing in Porcine Bowel Anastomosis

**DOI:** 10.1155/2020/5142797

**Published:** 2020-02-12

**Authors:** Hong Pan, Ping Keun Lam, See W. Tong, Kevin K. Leung, Anthony Y. Teoh, Enders K. Ng

**Affiliations:** ^1^Department of Surgery, Faculty of Medicine, The Chinese University of Hong Kong, Hong Kong, China; ^2^Department of General Surgery, Shanghai Jiahui International Hospital, Shanghai, China

## Abstract

**Objective:**

To evaluate the possible biological effect of allogenic mesenchymal stem cells (MSCs) combined with tissue fusion technology on the anastomosis.

**Methods:**

Sixteen pigs were divided into a 7 d group and 14 d group, each of which was further subdivided into an MSC-treated group and a control group. Five anastomoses per animal were established using LigaSure ForceTriad (Covidien, MA, USA), a tissue sealing system. Cell migration and tissue-specific differentiation potency, in addition to potential cytokine and genetic changes, were investigated.

**Results:**

There were no significant between-group differences in postoperative complications and anastomosis burst pressure. The number of proliferating cell nuclear antigen- (PCNA-) positive cells was significantly higher in the MSC-treated group as compared with that in the control group (*P* = 0.021). Labeled MSCs were found in the mucosal layer, villus, and lamina propria, as well as in the lamina muscularis mucosae, where they exhibited characteristics of smooth muscle cells.

**Conclusions:**

Grafted MSCs significantly promoted epithelial and connective cell proliferation and maintained their cell migration capacity and differentiation potential in the fused anastomotic tissues, without causing severe postoperative complications.

## 1. Introduction

The development of innovative surgical instruments has led to major advances in urological, colorectal, gynecological, and general surgery. Since the late 1990s, tissue fusion technology has led to the widespread application of LigaSure, a tissue sealing system, which has been proven to be safer than traditional vessel sealing methods, especially for vessels with diameters of <7 mm [1–7]. In the last decade, surgeons have attempted to use this sealing system in animal models and human clinical procedures for digestive gut sealing or anastomosis [8–15]. Unfortunately, these studies have reached no conclusions, irrespective of which type of system or prototype device based on tissue fusion technology was used.

In a preliminary study, we evaluated the feasibility and safety of tissue fusion technology in small bowel anastomosis using the LigaSure device in an *in vivo* porcine model. We achieved positive results, suggesting that this approach could be used to increase the safety of this type of anastomosis, without the risk of foreign materials. Biological techniques, especially those that promote cytokine production, provide a promising alternative to wound healing due to the absence of metal staples, glue, or sutures. The multipotent potential of stem cells has attracted much attention, leading researchers and clinicians to conduct *in vitro*, *ex vivo*, and *in vivo* experiments as well as some preclinical trials [16–22]. In terms of stem cell research, the most active domains are endocrine, orthopedics, neurology, and maxillofacial surgery. Many clinical trials in these areas have been approved, with stem cell research yielding promising results in terms of their wound healing effects [19, 20, 22–30].

This study is aimed at evaluating possible biological effects of allogenic mesenchymal stem cells (MSCs) for anastomosis, combined with tissue fusion technology.

## 2. Materials and Methods

### 2.1. MSC Preparation

Prior to stem cell transplantation, adipose-derived mesenchymal stem cells (ADMSCs) were harvested from the subcutaneous fatty tissues of pigs. The tissues were washed extensively with sterile phosphate-buffered saline (PBS) and treated with 0.1% collagenase (type I; Sigma-Aldrich) in PBS for 30 min at 37°C, with gentle agitation. After filtration through a 100 *μ*m mesh filter to remove debris, the filtrate was washed three times and suspended in Dulbecco's modified Eagle's medium, supplemented with 10% fetal bovine serum, 100 units/mL of penicillin, 100 *μ*g/mL of streptomycin, and 2 mM L-glutamine. The cultures were maintained in an incubator in a humidified atmosphere of 5% carbon dioxide ([Supplementary-material supplementary-material-1]).

To verify the differentiation potential of the ADMSCs, the cells were seeded at a density of 5,000 cells/cm^2^ and cultured in adipogenic, chondrogenic, and osteogenic differentiation culture media, according to the manufacturer's protocol (Invitrogen, Life Technologies™, USA). The differentiated adipocytes were stained with Oil Red O ([Supplementary-material supplementary-material-1]), the chondrocytes were stained with Alcian Blue ([Supplementary-material supplementary-material-1]), and the osteocytes were stained with Alizarin Red S stain ([Supplementary-material supplementary-material-1]) to identify intracytoplasmic lipids, extracellular glycosaminoglycan, and calcium deposits, respectively. All chemicals were purchased from Sigma-Aldrich Inc. (USA).

To label cultured ADMSCs with chemical dye for later tracking, 1 × 10^6^ DMSCs were labeled with 5 *μ*L of CM-Dil fluorescent dye (Invitrogen) for 20 min in an incubator at 37°C and then washed with PBS three times. After centrifugation at 1,500 rpm for 5 min, the cell pellets were resuspended in PBS, ready to be used.

The ADMSCs were grown for three to five passages and then harvested with 0.25% trypsin/ethylenediaminetetraacetic acid and characterized using a flow cytometer with a FACScan argon laser (BD Biosciences, San Jose, CA, USA). Briefly, the trypsinized cells were washed and centrifuged for 10 min at low speed, and 1 × 10^6^ cells were then fixed in 3% paraformaldehyde for 30 min at 4°C. The cells were then labeled with phycoerythrin-conjugated antibodies against CD166, CD13, CD29, CD44, CD105, CD45, and CD34 (Abcam Inc., Cambridge, U.K.). Isotype-matched negative controls were used to assess background fluorescence (Figures [Supplementary-material supplementary-material-1]). The data were analyzed using CellQuest software (Becton Dickinson).

### 2.2. Animals and Groups

Sixteen healthy pigs with weights of approximately 25 kg were used. Animal care was in accordance with the guidelines of the Department of Health of Hong Kong, and the animal experiments were approved by the Animal Experimentation Ethics Committee of the Faculty of Medicine of the Chinese University of Hong Kong.

The animals were divided into two groups: a 7 d group and a 14 d group (survived up to 7 or 14 d). Each group was then further subdivided into an MSC-treated group and a control group (no MSC treatment) (*n* = 4 in each subgroup).

### 2.3. Procedures

The animals were given a laxative and fasted for 24 h before the surgery. A laparotomy with a 5 cm incision in the epigastric region was performed under general anesthesia with endotracheal intubation and muscle relaxation. To create an anastomosis, the proximal jejunum was pulled out of the wound. Five anastomoses were created in each animal, and each anastomosis was approximately 30 cm apart. The anastomoses were established using LigaSure ForceTriad (Covidien, MA, USA) and were completed in a functional end-to-end format. The LigaSure working mode was set as 2 bars for energy output and single firing fusion according to our preliminary research results.

After the anastomoses had been established, the vehicle solution with or without MSCs was injected (*n* = 10) equidistantly along the anastomosis ring, with five injections administered at each side of the anastomotic junction using an insulin syringe (BD Ultra-Fine™, USA). The volume of each injection was 150 *μ*L, resulting in a total of 0.5 million MSCs for the MSC-treated group. When administering the injections, care was taken not to penetrate the bowel wall, and all injections were administered as precisely as possible in the subserosal layer. The abdominal incision was closed using continuous sutures. All the animals received antalgics to enhance the comfort of their resuscitation.

### 2.4. Postoperative Care

Posttreatment, the animals were housed in a room in the laboratory for 3 d to observe their recovery. The main parameters observed included temperature, weight change, mental status, food intake, and defecation. Venous injection of 4 mg/kg gentamicin was given for 2 d postsurgery. If an animal's temperature showed no sign of decline on the third day after the procedure, additional antibiotics were administered. Water was allowed from the first day, and a semiliquid diet was supplied from the second day. On the fourth day until the end of the observation period, the animals were transported to an animal facility on the university campus where they consumed a normal diet.

During the observation period, if the animal care staff reported that an animal exhibited clinical signs of bowel leakage or an intra-abdominal abscess, the animal was sacrificed. An autopsy was then performed for investigation of abnormal physical status.

### 2.5. Sample Collection

At the end of the observation period, the animals were weighed and subjected to general anesthesia. Relaparotomy was performed using an aseptic technique. Surgical site infections or abscesses, in addition to intra-abdominal collections or adhesions, ileus, and ascites, were recorded.

All anastomoses were checked. A severe abscess or dense fibrous adhesion was considered a sign of leakage. Each intact anastomotic site was harvested by transecting 5 cm proximal and distal to the anastomosis. The samples were incubated in iced saline, and burst pressure was assessed and recorded by a device and computer software of Handheld Meter's Data Logger (version 2.0). Once the pressure data and burst positions were recorded, the harvested samples were incised along the longitudinal axis and inspected for microulcers, microabscesses, or other lesions on the mucosal surface. Tissue samples were obtained from intact anastomoses for cryopreservation in liquid nitrogen for Western blot analysis, polymerase chain reaction (PCR) arrays, and paraffin embedding using buffered formalin in immune-histological staining. After the sample collection, all the animals were euthanized.

### 2.6. Hematoxylin and Eosin (H&E) Staining and Trichromatic Staining

One anastomotic sample from each animal was paraffin-embedded and incised into 4 *μ*m sections (4 anastomotic samples per subgroup). Masson's trichromatic staining protocol was used with Mayer's hematoxylin solution (Vector Hematoxylin QS, H-3404-100) according to routine immunohistochemical procedures. The concentration of the primary antibody for proliferating cell nuclear antigen (PCNA) (sc-56, Santa Cruz Biotechnology, Inc., USA) was diluted at 1 : 5,000, and CD31 (sc-1506, Santa Cruz Biotechnology, Inc., USA) was diluted at 1 : 50. The number of PCNA-positive cells (dark brown) and newly formed vessels (brown endothelial membrane) was counted under a 200x visual field by two Ph.D. students independently in a double-blind manner. The cells were counted in three different fields near each anastomotic junction in each anastomosis. For PCNA, two epithelial regions beside the fusion line and one region in the fusion area were included. All three CD31-counting fields were located in the fusion area.

### 2.7. Immunofluorescent Staining

Immunofluorescent staining for MSC tracking in the bowel wall relied on the CM-Dil fluorescent dye, which reflected a red color under a laser. The differentiation potential of MSC cells was investigated using anti-alpha smooth muscle actin (1 : 100; ab5694, Abcam, USA) and anti-pan cytokeratin (1 : 50; ab7753, Abcam, USA). The nucleus was stained with 4,6-diamidino-2-phenylindole (Life Technologies™, USA). Images of stained slides were captured by a Leica® DM40008LED DFC450C camera and Leica LAS-AF Lite software. Images were analyzed by ImageJ2x software (Wayne Rasband, National Institutes of Health, USA).

### 2.8. Western Blot

Tissue samples kept in liquid nitrogen were routinely processed for lysate preparation, protein loading, electrophoresis, transfer, and staining. Actin was used as the loading control. The protein loading volume was 30 *μ*g in each well. The primary antibody concentration and exposure time of the blot were as follows: vascular endothelial growth factor (VEGF; 1 : 300, 3 min; sc-507, Santa Cruz Biotechnology, Inc., USA), fibroblast growth factor-basic (FGF2; 1 : 300, 3 min; sc-1360, Santa Cruz Biotechnology, Inc., USA), cluster of differentiation 31 (CD31; 1 : 200, 30 s; sc-1506, Santa Cruz Biotechnology, Inc., USA), and actin (1 : 5,000, 2 min; sc-1615, Santa Cruz Biotechnology, Inc., USA). All the secondary antibody concentrations were 1 : 2,000. As the molecular weights of VEGF (42 kDa) and actin (43 kDa) were similar, Restore™ Western Blot Stripping Buffer (21059; Thermo Fisher Scientific, Inc., USA) was used for actin blotting, and the washing time in the stripping buffer was 15 min. The blots were scanned and analyzed using software (iBright CL1500 Imaging System; Thermo Fisher Scientific, Inc., USA). The ratio of the target protein to actin in each sample in each group was compared 7 d and 14 d postsurgery.

### 2.9. PCR Array

In the 7 d group, six anastomotic samples were collected from three MSC-treated animals and three control animals. The RNA extraction was conducted using the RNeasy FFPE kit (QIAGEN, Germany) according to the manufacturer's instruction. For cDNA synthesis, 0.5 *μ*g of extracted RNA from the anastomotic sample in the genomic DNA elimination mix was used. The mixture was incubated at 42°C for 5 min and then cooled on ice for 1 min. Reverse transcription mix was then added at an equal volume of genomic DNA elimination mix and incubated at 42°C for 15 min. The reaction was then immediately stopped by incubating at 95°C for 5 min.

The real-time PCR was performed using an RT^2^ Profiler and QuantStudio™ 12K Flex system (Applied Biosystems, Life Technologies, USA). The RT^2^ Profiler PCR array for wound healing in the animals was conducted in triplicate for each sample. Analysis of gene expression was conducted using online software provided by QIAGEN (http://pcrdataanalysis.sabiosciences.com/pcr/arrayanalysis.php). The expression of each gene was normalized to that of the housekeeping genes ACTB, B2M, GAPDH, HPRT1, and RPL13A. The fold changes of mRNA in anastomotic tissue in the MSC-treated group were calculated using the 2(-*ΔΔ*Ct) method. Changes of potential signaling pathways and interactions of intestinal wound healing-related genes were analyzed using the QIAGEN software package (http://gncpro.sabiosciences.com/gncpro/gncpro.php).

### 2.10. Statistical Analysis

SPSS Statistics, version 19.0.0 (IBM, Armonk, NY, USA), software was used for data analysis. The Mann–Whitney *U* test was used to compare differences between groups. Student's *t*-test was conducted to analyze the PCR array results. *P* values of <0.05 were considered statistically significant.

## 3. Results

### 3.1. Local Injection of MSCs Did Not Influence Clinical Outcomes

All the animals were sacrificed at the time of the second laparotomy at the end of the observation period, except one. This animal showed a reduced level of activity and obvious marasmus on d 9 postsurgery, and it was sacrificed on d 10.

Table 1 shows the postoperative complications in the animals. High fever was defined as the temperature higher than 39°C in the first 3 d postsurgery. Marasmus was defined as a weight loss of more than 5 kg on the morning of sacrifice. There was no significant difference between the MSC-treated group and the control group in terms of a high fever, marasmus, bowel distention, ileus, ascites, surgical site infection, and hemafecia.

Severe abscesses close to the anastomoses were considered evidence of leakage, whereas anastomoses without severe adhesions or obvious abscesses around the anastomotic sites were considered intact (safe anastomoses). Leakage occurred in one anastomosis in one animal in both the MSC-treated group and the control group at the 7 d timepoint. At the 14 d timepoint, there were two cases of anastomotic leakage in the control group and one case in the MSC-treated group (Table 1). Therefore, the leakage rate was 5% in the MSC-treated group and 7.5% in the control group.

### 3.2. Local Injection of MSCs Did Not Affect Burst Pressure

**Figure 1:**
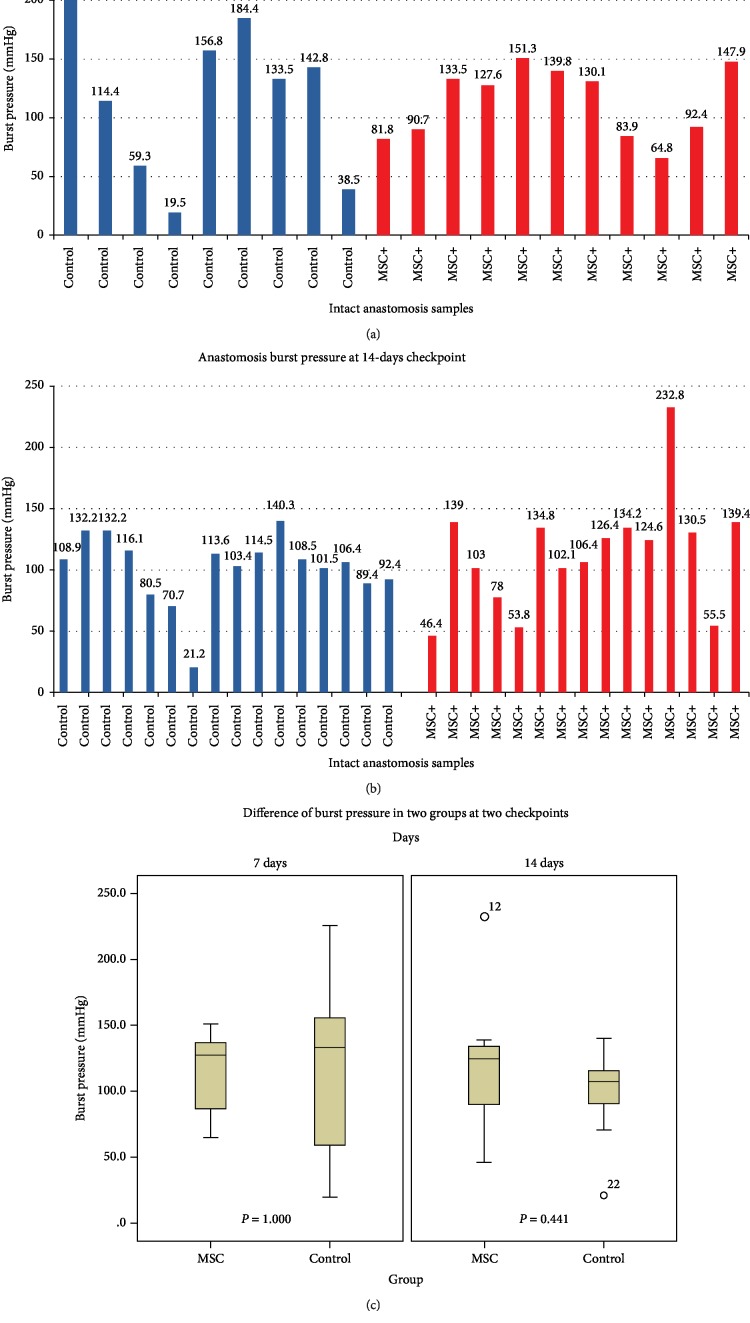
Comparison of burst pressures between the two groups. (a) Burst pressures of intact anastomoses at the 7 d timepoint in the MSC-treated group (*n* = 11) and the control group (*n* = 9). (b) Burst pressures of intact anastomoses at the 14 d timepoint in the MSC-treated group (*n* = 15) and the control group (*n* = 16). (c) Comparison of burst pressures between the two groups.

Due to the physiological wound healing process, anastomosis dissection and sample collection were more difficult in the 7 d group because of extensive intra-abdominal adhesion between the loops as compared with that in the 14 d group. For the same reason, analysis of burst pressure was more difficult in the 7 d postsurgery group, with only 11 of 19 intact anastomotic samples obtained from the MSC-treated group and 9 of 19 samples obtained from the control group for burst pressure assessments (Figure 1(a)). At the 14 d timepoint, 15 of 19 intact anastomotic samples were obtained from the MSC-treated group, and 16 of 18 intact samples were obtained from the control group (Figure 1(b)). Due to the data distribution, we used the median and interquartile range (IQR) to represent burst pressure in each group at different timepoints. At the 7 d timepoint, burst pressure was 127.60 mmHg (83.90–139.80) in the MSC-treated group and 134.34 mmHg (48.90–170.60) in the control group. At the 14 d timepoint, burst pressure was 124.60 mmHg (78.00–134.80) in the MSC-treated group and 107.45 mmHg (90.15–115.70) in the control group (Figure 1(c)). The Mann–Whitney *U* test revealed no significant between-group difference in burst pressure at the two timepoints (*P* = 1.000 and *P* = 0.441, respectively).

### 3.3. Grafted MSCs Promoted Epithelial and Connective Cell Proliferation

H&E staining and trichromatic staining were performed to examine morphological changes in the anastomotic sites. As shown by the results of H&E staining (100x), no obvious morphological differences were observed in the subserosa adjacent to the anastomotic rings in the MSC-treated group as compared with those in the control group ([Supplementary-material supplementary-material-1]). Total reepithelialization was observed in the MSC-treated group but not in the control group at the 7 d timepoint. At the 14 d timepoint, the extent of reepithelialization at the anastomotic junctions was similar in the two groups. At both timepoints, neovascularization and inflammatory cell infiltration in the anastomoses in the MSC-treated group and the control group appeared similar.

The trichromatic staining images showed no structural differences in the arrangement of collagen fibers, which filled the gap between the two lateral extremities of the muscular layers, between the two groups. Blue-stained fibers occupied a dominant portion of connective tissue, which sustained the integrity of the anastomotic rings 2 wk after the procedure (Figures [Supplementary-material supplementary-material-1] and [Supplementary-material supplementary-material-1]).

### 3.4. Grafted MSCs Promoted PCNA and CD31 Expression

**Figure 2:**
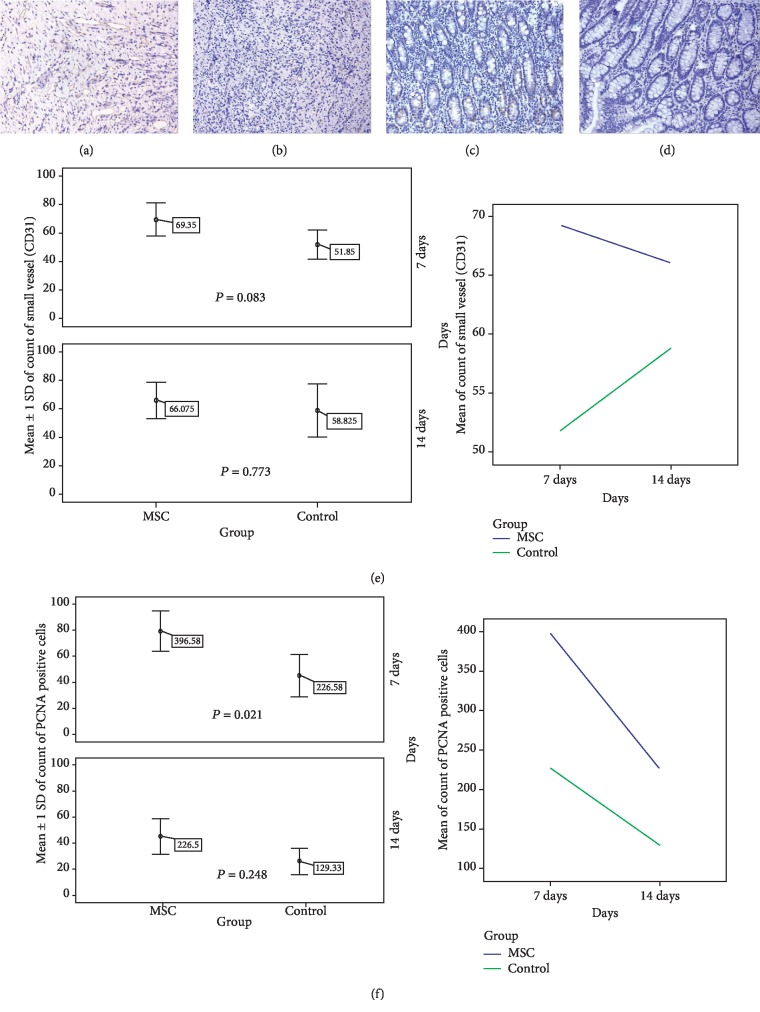
Between-group comparison of PCNA and CD31. (a) IHC image of CD31 (ID PH34, MSC-treated group, 7 d, 200x). (b) IHC image of CD31 (ID PH36, control group, 7 d, 200x). (c) IHC image of PCNA (ID PH37, MSC-treated group, 7 d, 200x). (d) IHC image of PCNA (ID PH36, control group, 7 d, 200x). (e) Between-group comparison of the number of CD31-stained small vessels (*n* = 4). (f) Comparison of the number of PCNA-positive cells between the two groups (*n* = 4).

The number of PCNA-positive cells and small vessels, based on immunohistochemical staining of PCNA and CD31, respectively, was counted in one intact anastomotic sample from each animal (Figures 2(a)–2(d)). The number of PCNA-positive cells and small vessels in the MSC-treated group was greater than that in the control group at both the 7 d and 14 d timepoints (Table 2). However, at the 7 d timepoint, only the number of PCNA-positive cells was significantly higher in the MSC-treated group as compared with that in the control group (*P* = 0.021) (Figures 2(e) and 2(f)).

### 3.5. Verification of MSC Cell Migration Capacity and Differentiation Potential in the Fused Anastomotic Tissue

**Figure 3:**
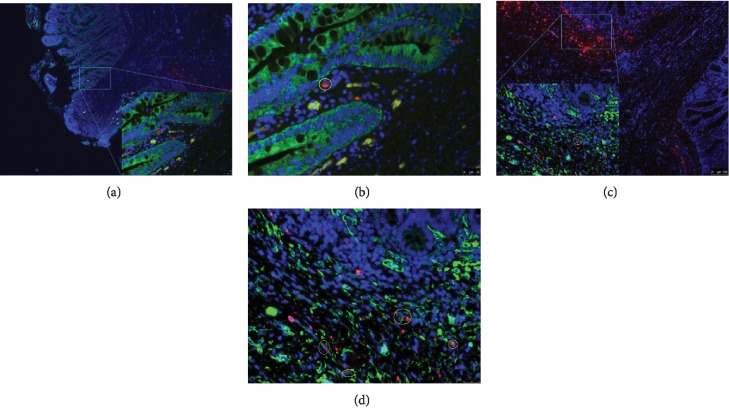
MSC tracking and differentiation. (a) Tracking of grafted MSCs (50x) on d 14. (b) Tracking of grafted MSCs (400x) on d 14, showing a zoomed-in view of (a). (c) Differentiation of smooth muscle cells from grafted MSCs (50x) on d 14. (d) Differentiation of smooth muscle cells from grafted MSCs (400x) on d 14, showing a zoomed-in view of (c). Red: CM-Dil fluorescent dye. Green: anti-alpha smooth muscle actin in (a, b) and pan cytokeratin in (c, d). Blue: 4,6-diamidino-2-phenylindole.

The cell migration of the injected MSCs was examined by immunofluorescent staining. MSCs were detected in the mucosal layer, as well as in the villus, where reepithelialization took place adjacent to the anastomotic junction (Figures 3(a) and 3(b)). The lamina propria seemed to be the most distant layer of MSC cell migration, as CM-Dil fluorescent dye-marked epithelial cells that were supposed to be differentiated from injected MSCs were not found. Anti-alpha smooth muscle actin staining revealed some cells (marked with CM-Dil fluorescent dye) in the lamina muscularis mucosae. These cells also exhibited characteristics of smooth muscle cells (i.e., a positive reaction with antigens), indicating that these smooth muscle cells were differentiated from the injected MSCs (Figures 3(c) and 3(d)).

### 3.6. MSCs Did Not Influence the Expression of CD31, VEGF, and FGF2

**Figure 4:**
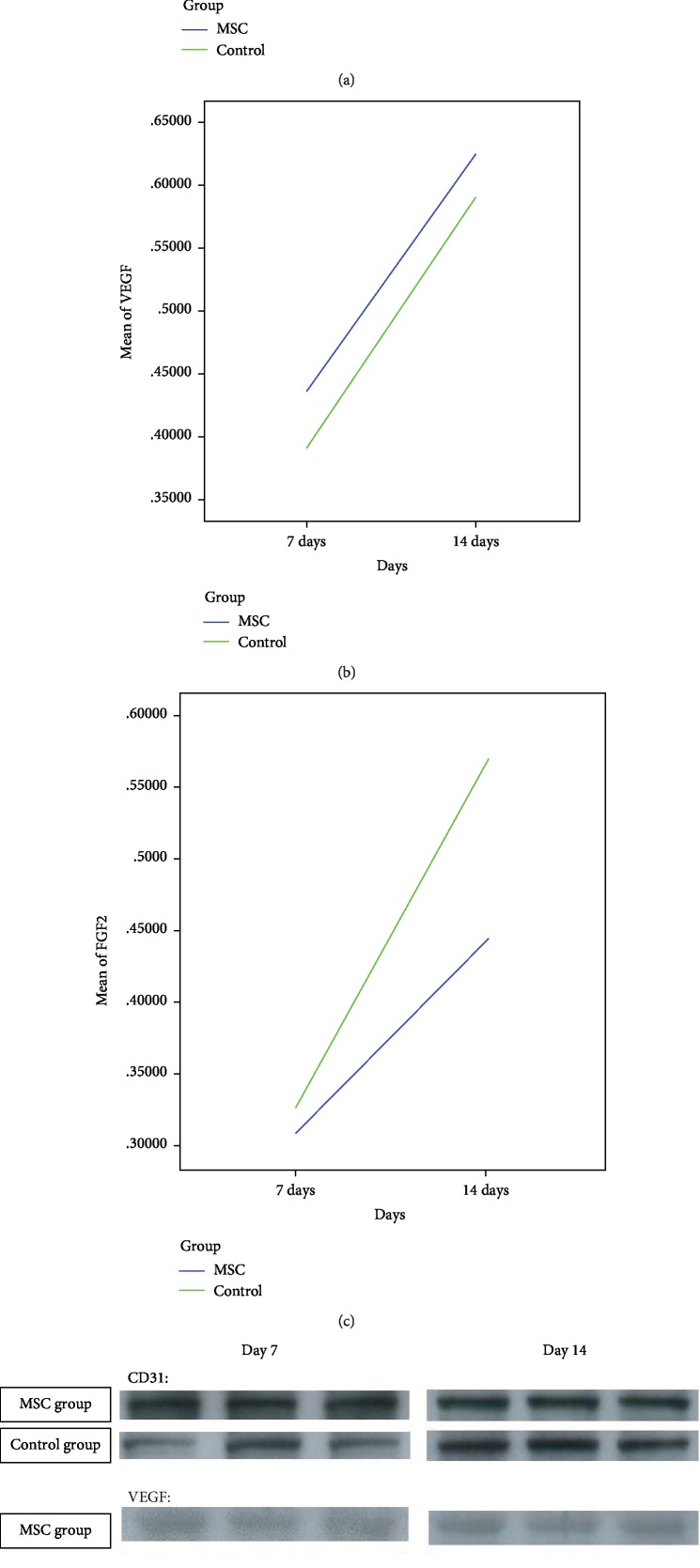
Western blot analysis of proteins involved in the wound healing process. (a) Tendency of change in CD31 expression in anastomotic tissue (*n* = 4). (b) Tendency of change in VEGF expression in anastomotic tissue (*n* = 4). (c) Tendency of change in FGF2 expression in anastomotic tissue (*n* = 4). (d) Representative images for the Western blot.

Three proteins involved in the wound healing process (i.e., CD31, VEGF, and FGF2) were semiquantitatively analyzed using a Western blot. As shown in Table 3, there was no significant difference in the expression of these proteins in the MSC and control groups at the 7 d and 14 d timepoints, as shown by the Mann–Whitney *U* test. The tendency of expression change in each protein in anastomotic tissues is shown in Figure 4.

### 3.7. MSCs Lowered the Expression of Numerous Wound Healing-Related Genes in Anastomotic Tissues

The mRNA profiles and levels in anastomotic tissues from three animals in the MSC-treated group and three animals in the control group were determined by PCR analysis. Eighty-four key genes critical for the wound healing response were identified using the RT^2^ Profiler PCR array ([Supplementary-material supplementary-material-1]). These genes were divided into several groups, as follows: (1) extracellular matrix (ECM) structural constituents (*COL14A1*, *COL1A1*, *COL1A2*, *COL3A1*, *COL4A3*, *COL5A2*, *COL5A3*, *DCN*, *EDN1*, and *VTN*), (2) ECM remodeling enzymes (*CTSK*, *F13A1*, *FGA*, *CTSG*, *PLAUR*, *F3*, *MMP1*, *MMP2*, *MMP3*, *MMP7*, *MMP9*, *PLAT*, *PLAU*, *PLG*, and *SERPINE1*), (3) cell adhesion molecules (*CDH1*, *ITGA2*, *ITGA3*, *ITGA4*, *ITGA5*, *ITGA6*, *ITGAV*, *ITGB1*, *ITGB3*, *ITGB5*, *ITGB6*, and *TNC*), (4) cytoskeleton regulators (*ACTA2*, *ACTC1*, *RAC1*, and *TAGLN*), (5) inflammatory cytokines and chemokines (*CCL2*, *CD40LG*, *CXCL11*, *CXCL12*, *CXCL2*, *IFNG*, *IL1A*, *IL10*, *IL1B*, *IL2*, and *IL4*), (6) growth factors (*ANGPT1*, *CCN2*, *CSF2*, *CSF3*, *EGF*, *FGF2*, *FGF7*, *HBEGF*, *IGF1*, *LOC100525086*, *MIF*, *TGFA*, *TGFB1*, *TNF*, and *VEGFA*), (7) TGF*β*/BMP signaling pathway molecules (*SMAD3*, *TGFB1*, *TGFB2*, *TGFB3*, and *TGFBR3*), (8) WNT signaling pathway molecules (*CTNNB1*, *LOC100627044*, and *WNT5A*), (9) kinases (*AKT1*, *EPHB2*, *MET*, *MYLK*, and *PTEN*), (10) cell surface receptors (*EGFR*, *EPHB2*, *IL6ST*, *MET*, and *TGFBR3*), and (11) other signal transduction genes (*PTGS2*, *PTGS1*, and *STAT3*).

**Figure 5:**
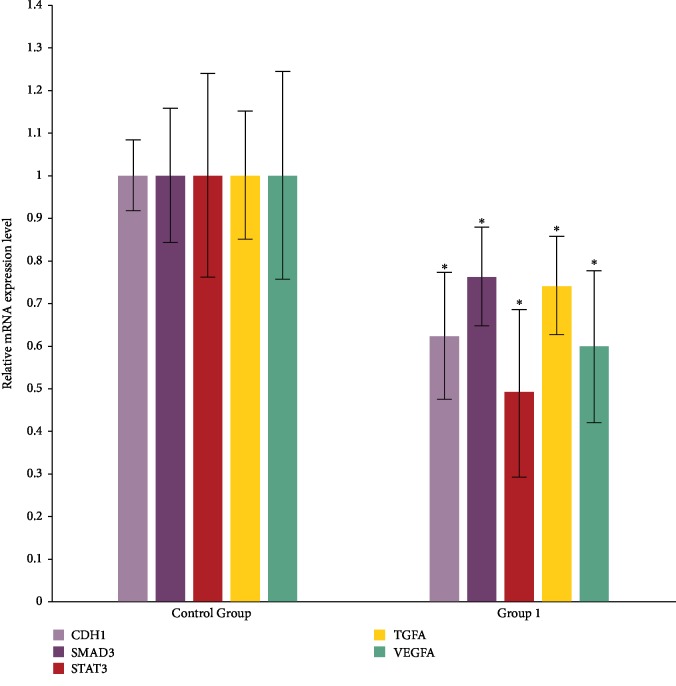
Five genes of interest showed significant downregulation in anastomotic tissues in the MSC-treated group (*n* = 3) as compared with that in the control group (*n* = 3). ^∗^*P* < 0.05 vs. control group.

Among these 84 genes of interest, only ten genes were upregulated in the MSC-grafted samples: *CD40LG*, *COL4A3*, *CXCL11*, *FGA*, *IFNG*, *IL4*, *CTSG*, *PLG*, *ALPL*, and *PTEN*. However, there was no statistically significant between-group difference in the expression of these genes. In contrast, the expression of the other 75 genes decreased in the MSC-grafted samples as compared with that in the control samples. Of these, the downregulation of five genes showed statistical significance (Figure 5). The potential signaling pathways are shown in [Supplementary-material supplementary-material-1].

## 4. Discussion

In this study, local injection of allogeneic MSCs at sites of tissue-fused anastomoses did not influence clinical outcomes, including the leakage rate and burst pressure. The grafted MSCs significantly promoted epithelial and connective cell proliferation. MSC cell migration capacity and differentiation potential in fused anastomotic tissue were verified. Local injection of allogenic MSCs may lead to the downregulation of the expression of numerous wound healing-related genes in anastomotic tissues 7 d post graft.

This is the first study to investigate the healing capacity of MSCs in the treatment of small bowel anastomoses using electronic tissue fusion technology in an *in vivo* animal model. Based on our preliminary experiments, we expected better clinical or laboratory outcomes in the present study as compared with those obtained using simple tissue fusion technology [31, 32]. Given the nature of the tissue healing process and findings in the literature on grafted stem cell survival, we chose 7 and 14 d as the timepoints postsurgery. After 7 d, the effects of locally injected stem cells, especially on burst pressures of anastomoses, would be expected to be much more obvious than those after 14 d when adhesions are well established. However, in the early postsurgery period (i.e., 7 d), it may be more difficult to detect stem cell differentiation in the anastomotic site. Clinically, anastomosis leakage occurs most frequently 2 wk postsurgery. Therefore, we observed cell differentiation of grafted stem cells at the end of 14 d.

In previous studies that employed rat models, the number of grafted stem cells around gastric incisions or colonic anastomoses ranged from 1 to 10 × 10^6^ cells (0.5 m) [33–36]. In some clinical trials, the concentration of administered stem cells was approximately 1 × 10^7^/mL in the treatment of gut fistulas with autologous MSCs [37–40]. In our study, 0.5 million MSCs were injected, leading to an acceptable leakage rate in each animal in which five anastomoses were created. Considering that anastomosis leakage cannot be verified in clinical cases, tissue fusion technology, combined with stem cells, provides an alternative treatment method that can ensure anastomosis safety.

During the dissection of anastomoses from sacrificed animals, we were unable to collect samples from all the anastomoses due to intra-abdominal adhesions. Burst pressure assessments of intact anastomoses showed no significant difference between the two groups. Previous studies that used a porcine model reported burst pressures ranging from 10 to 70 mmHg [12–14]. As compared with these pressures, those found in our study were much higher, especially at the 14 d timepoint. The discord in the findings may be due to the presence of pervasive adhesions in loops increasing the tensile strength. In some cases, not only the condition of the anastomosis ring but also the adhesions around the anastomosis influence the risk of leakage. We speculate that for swine, local injection of stem cells has little observable effects on burst pressure, relative to other systemic factors, such as health and nutritional condition.

We also found no obvious differences in the architecture of the anastomotic sites in the two groups. The distance between two extremities of the muscle layer and the process of epithelium coverage depended on its category of contact surface rather than on the MSC injection. However, early in the healing process (i.e., 7 d postsurgery), we detected a significantly higher number of proliferating cells in the MSC-treated group as compared with that in the control group. These actively proliferating cells included epithelial cells and cells of connective tissues, which could be fibroblasts, smooth muscle cells, or endothelial cells of new capillaries. In addition, at both timepoints, higher numbers of PCNA-positive cells and small vessels were observed in the MSC-treated group as compared with those in the control group at both timepoints, although the findings were not statistically significant. From 7 to 14 d, the number of vessels decreased in the MSC-treated group but increased in the control group. In addition, there were larger diameter vessels in the MSC-treated group. Larger vessels are more advantageous for oxygen and nutrition transportation, which is beneficial in the early period of anastomosis healing. These results were verified by the Western blot analysis of CD31, which showed increased CD31 staining in the MSC-treated group as compared with that in the control group. In addition, VEGF expression in anastomotic tissue in the MSC-treated group was higher than that in the control group at both timepoints, although the difference was not statistically significant. This finding was contrary to our hypothesis about the tissue levels of FGF2. This result is difficult to interpret in the absence of information on the role of cytokines in the healing process.

Only a few studies using *in vivo* animal models have provided evidence on grafted stem cell differentiation capacity. Our study demonstrated the short-distance migration capacity of allogeneic MSCs from the subserosal layer to the submucosal layer. These cells cannot penetrate the smooth muscle layer in the small bowel wall, and the only possible route in which they could migrate to the submucosa is the gap between two extremities of the muscle layer. Based on our immunofluorescent results, we speculate that the grafted stem cells differentiated into smooth muscle cells during this migration process. A microenvironment of low oxygen and denatured protein components may favor stem cell survival and stem cell differentiation [41, 42]. In the present study, in terms of the migration distance, the most distant stem cells were located at the axis of the villus adjacent to each anastomotic site. However, none of these stem cells differentiated into epithelial cells. It should be mentioned that Dil can leak from dead cells [43]; therefore, we need to exclude the possibility that CM-Dil-marked cells are derived from dead cells in our future work.

As gene changes occur in advance of protein expression, the samples obtained at the 7 d timepoint in the two groups were employed in the PCR array analysis. The results demonstrated that the expression of the majority of genes of interest decreased in the anastomotic tissues in the MSC-treated group. The decrease in the expression profile of wound-related genes was possibly due to the adding portion of the injected MSCs or a decrease in intrinsic pig cells. Five genes, including *VEGF*, exhibited a significant fold change, and these genes were considered as functional genes encoding proteins involved in epithelial or blood vessel endothelial proliferation. The expression of most fibroblast growth factor-encoding genes, such as *FGF2*, was reduced. The Western blot results showed that the protein level of FGF2 was consistent with its mRNA level, whereas the reverse was the case for VEGF. We suspect that negative feedback mechanisms in the process of gene transcription may suppress mRNA levels in response to abundant protein (e.g., VEGF) synthesis. For example, as shown in the potential signaling pathway network shown in [Supplementary-material supplementary-material-1], *STAT3* and *VEGFA* were not only coexpressed but also subjected to feedback suppression through the IL6 signaling pathway. However, *VEGF* also appeared to be strongly upregulated by two other genes, *SMAD3* and *CDH1*. These findings suggest that VEGFA is critical for endothelial cell differentiation and cooperates with epidermal growth via STAT3 and stem cell differentiation through the TGF*β*/SMAD signaling pathway.

## 5. Conclusions

Local injection of allogenic MSCs at sites of small bowel anastomoses did not influence clinical outcomes, including the anastomosis leakage rate and burst pressure. Grafted MSCs significantly promoted epithelium and connective cell proliferation through their paracrine effects in anastomotic tissue, which was fused using high-frequency tissue fusion technology. The cell migration capacity and differentiation potential of the MSCs in the fused anastomotic tissues were verified. Local injections of allogenic MSCs may lead to decreased expression of numerous wound healing-related genes in anastomotic tissues 7 d post graft, suggesting the need for further investigations of gene expression at an earlier postoperative stage.

## Figures and Tables

**Table 1:** Postoperative complications in the animals.

Complications	7 ds-MSC	7 ds-control	14 ds-MSC	14 ds-control
High fever	3^#^	2^^^	/	1
Marasmus	/	/	1^∗^	/
Bowel distention	1^#^	/	/	/
Ileus	/	/	1^∗^	/
Ascites	/	1	/	/
Surgical site infection	/	1^^^	/	/
Hemafecia	/	/	/	/
Anastomosis leakage	1	1	1	2

^∗^Obvious weight loss 9 d postsurgery, despite some food intake and stool formation. A postmortem examination 10 d postsurgery showed an incomplete ileus caused by an adhesion around the first upstream anastomosis. ^#^One animal with a high fever showed light ileum distension, with abundant air, as well as diarrhea. ^^^One animal developed a localized abscess in the abdominal surgical site.

**Table 2:** IHC staining of the anastomosis junction (mean ± SD).

	7 days		14 days	
	Control	MSC	Control	MSC
New vessels (CD31)	51.85 ± 10.26	69.35 ± 11.62	58.83 ± 18.77	66.08 ± 13.01
PCNA^+^ cells	226.58 ± 80.55	396.58 ± 76.82^∗^	129.33 ± 50.30	226.50 ± 68.20

^∗^ *P* = 0.021 vs. control group (7 days).

**Table 3:** Western blot analysis of three proteins involved in the wound healing process (mean ± SD).

	7 days		14 days	
	Control	MSC	Control	MSC (*n* = 19)
CD31	1.328 ± 0.228	1.266 ± 0.175	1.456 ± 0.548	1.610 ± 0.610
VEGF	0.391 ± 0.093	0.436 ± 0.115	0.591 ± 0.146	0.625 ± 0.237
FGF2	0.326 ± 0.154	0.309 ± 0.179	0.570 ± 0.229	0.445 ± 0.356

## Data Availability

The analyzed data sets generated during the study are available from the corresponding author on reasonable request.

## Supplementary Materials

Supplementary Materials Figure S1: mesenchymal stem cell preparation. A. Morphology of adherent ADMSCs. B. Differentiated adipocytes stained with Oil Red O. C. Differentiated chondrocytes stained with Alcian Blue. D. Differentiated osteocytes stained with Alizarin Red S. E-H. Identification of cellular markers of ADMSCs with flow cytometry. Figure S2: morphology of the anastomotic site. A. H&E staining image of the anastomotic site (control group, 50x). B, C. Trichromatic staining images of the anastomotic site (control group, 50x and 400x, respectively). Figure S3: magnitude of 84 genes related to intestinal wound healing in the MSC and control groups. Figure S4: potential signaling pathway network formed by the 5 significantly downregulated genes in this study.
