# Supplementary material for: Mesenchymal Stem Cells Combined with Tissue Fusion Technology Promoted Wound Healing in Porcine Bowel Anastomosis
Source: Stem Cells Int. 2020 Feb 12;2020:5142797. doi: 10.1155/2020/5142797 (PMC7038387; doi:10.1155/2020/5142797)
Supplement: Supplementary Materials — Figure S1: mesenchymal stem cell preparation. A. Morphology of adherent ADMSCs. B. Differentiated adipocytes stained with Oil Red O. C. Differentiated chondrocytes stained with Alcian Blue. D. Differentiated osteocytes stained with Alizarin Red S. E-H. Identification of cellular markers of ADMSCs with flow cytometry. Figure S2: morphology of the anastomotic site. A. H&E staining image of the anastomotic site (control group, 50x). B, C. Trichromatic staining images of the anastomotic site (control group, 50x and 400x, respectively). Figure S3: magnitude of 84 genes related to intestinal wound healing in the MSC and control groups. Figure S4: potential signaling pathway network formed by the 5 significantly downregulated genes in this study. [file 5142797.f1.pdf]

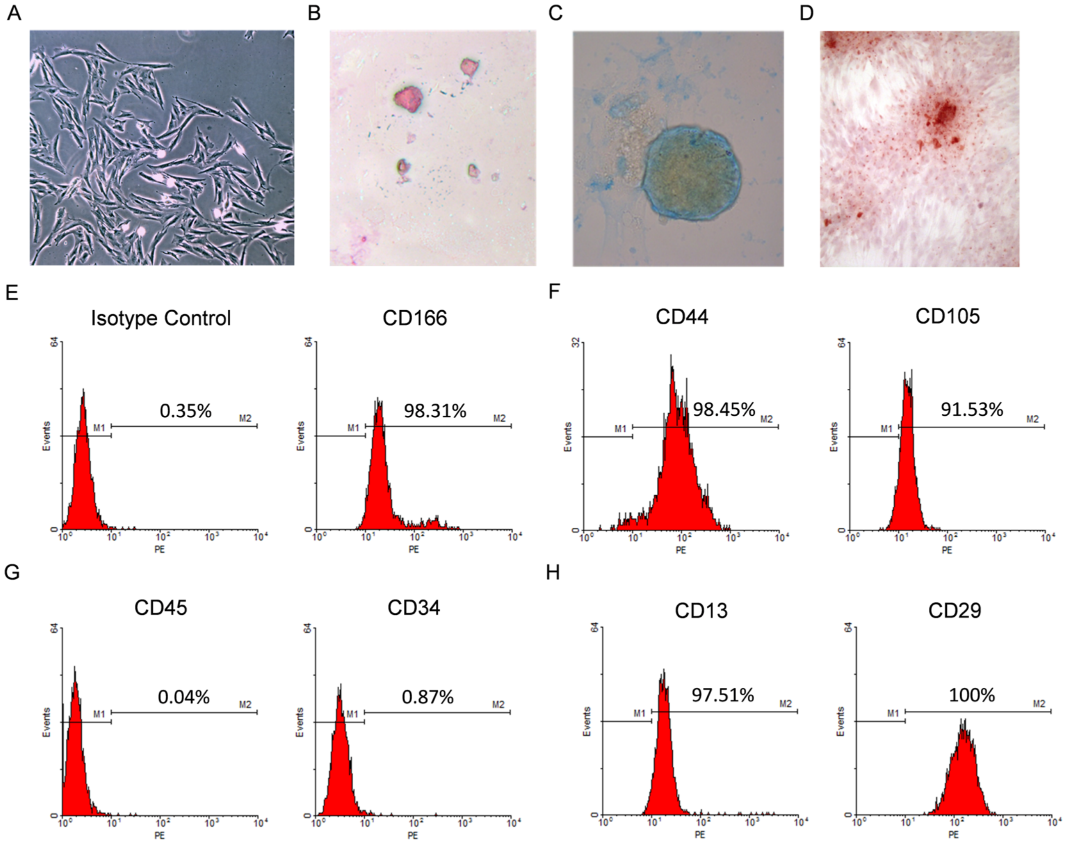

Figure S1. MSC preparation. A. Morphology of adherent ADMSCs. B. Differentiated adipocytes stained with Oil Red O. C. Differentiated chondrocytes stained with Alcian Blue. D. Differentiated osteocytes stained with Alizarin Red S. E–H. Identification of cellular markers of ADMSCs with flow cytometry.

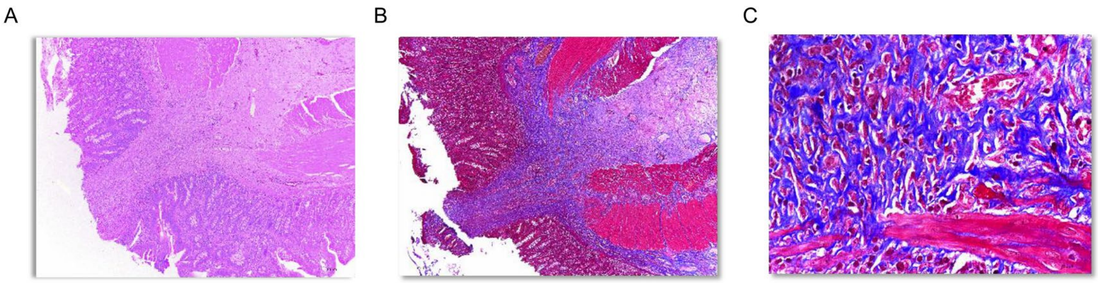

Figure S2. Morphology of an anastomotic site. A. H&E staining image of an anastomotic site (control group, 50×). B, C. Trichromatic staining images of an anastomotic site (control group, 50× and 400×, respectively).

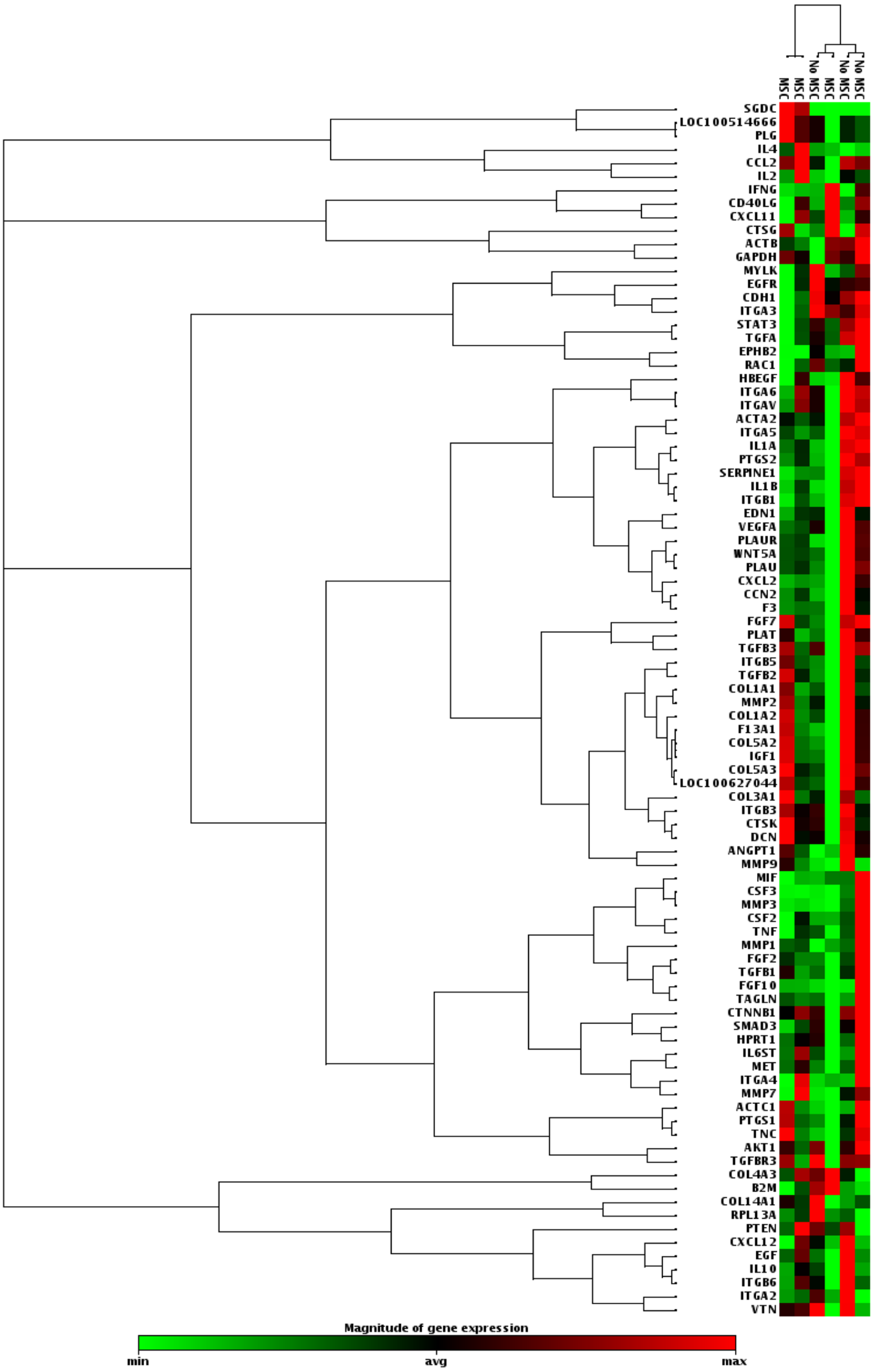

Figure S3. Expression of 84 genes related to intestinal wound healing in the MSC and control groups.

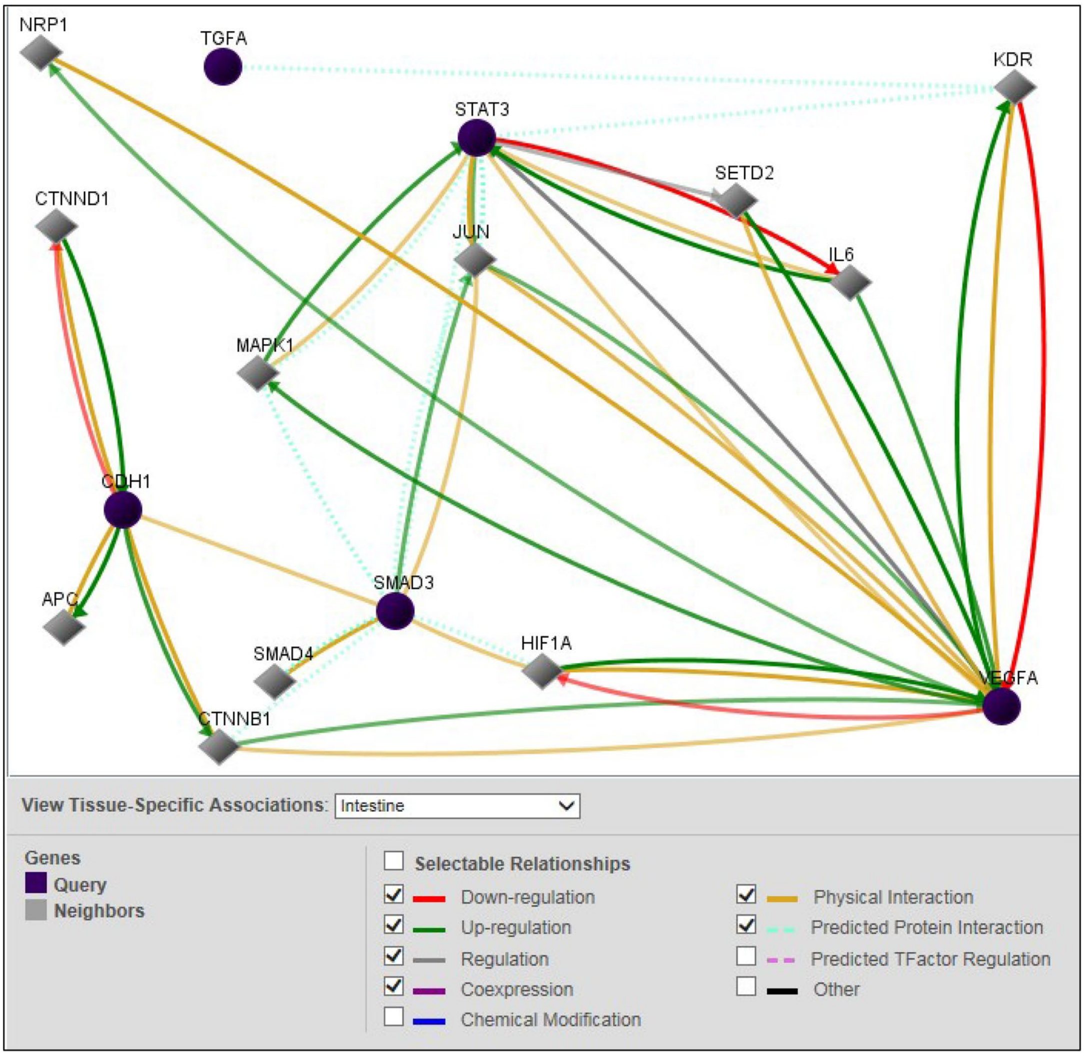

Figure S4. Potential signaling pathway network formed by five significantly downregulated genes.
